# 肠道菌群与肺癌的相关性

**DOI:** 10.3779/j.issn.1009-3419.2020.101.39

**Published:** 2020-10-20

**Authors:** 俊 滕, 艳芬 赵, 云宁 姜, 琦 王, 永生 张

**Affiliations:** 1 100029 北京，北京中医药大学研究生院 Graduate School, Beijing University of Chinese Medicine, Beijing 100029, China; 2 100078 北京，北京中医药大学附属东方医院呼吸科 Department of Respiratory, Dongfang Hospital, Beijing University of Chinese Medicine, Beijing 100078, China

**Keywords:** 肠道菌群, 肺肿瘤, 代谢, 炎症, 免疫反应, Gut microbiota, Lung neoplasms, Metabolism, Inflammation, Immune response

## Abstract

基因-环境相互作用是癌症易感性和进展的基础。人体长期暴露于各种微生物所构成的微环境中并受其影响，如肠道菌群所构成的微环境。肺癌患者中某些肠道微生物的相对丰度与对照组相比存在显著差异。这些研究表明，肠道菌群可能通过某些途径与肺癌相关联。同时，与我们所暴露的外部环境相比，肠道菌群相对而言是易控制的环境变量，因为它们在个体内是高度可量化和相对稳定的。正如某些靶向肠道菌群的肺癌的诊断、干预、治疗的措施已在临床取得一定效果。在这篇综述中，我们主要讨论了肠道菌群通过某些途径，如通过调控代谢、炎症以及免疫反应影响肺癌的发生发展以及治疗。根据当前的研究进展推断：针对肠道菌群的研究或将是肺癌进行精准和个性化医疗的有效途径。

## Abstract (en)

Gene-environment interactions underlie cancer susceptibility and progression. The human body is exposed to and affected by the microenvironment seiscasts of various microorganisms and their metabolites, such as the microenvironment of gut microbiota. The relative abundance of some intestinal microbes in lung cancer patients was significantly different from that in the control group. These studies suggest that gut microbiota may be associated with lung cancer through some ways. At the same time, gut microbiota is relatively manageable environmental variables compared to the external environment we are exposed to, as they are highly quantifiable and relatively stable in the individual. Just as some measures of diagnosis, intervention and treatment of lung cancer targeting gut microbiota have achieved some results in clinical practice. In this review, we mainly discuss the role of gut microbiota and its metabolites in the progression and treatment of lung cancer through certain ways, such as regulation of metabolism, inflammation, and immune response. Finally, based on current research progress, it is inferred that research on gut microbiota may be an effective approach to the precise and personalized medical treatment of lung cancer.

由于遗传学研究工具的出现和过去十数年的宏基因组革命，我们得以描述来自身体不同部位的微生物群的组成和功能，特别是对于寄生于人体肠道中的微生物有了更为深入的了解，它们既是宿主新陈代谢的关键贡献者，也被发现与多种疾病的发生发展存在潜在的联系。研究发现，以肥胖^[1]^为代表的人体代谢紊乱以及如哮喘、阿尔茨海默病、多种恶性肿瘤^[2-4]^等疾病的发生风险与肠道菌群组成或功能的紊乱关系密切。

## 1 肠道菌群概述

肠道菌群在人体肠道中有多种基本功能，如将不可消化的食物成分发酵成可吸收的代谢物、合成必需的维生素、去除有毒化合物、抵御病原体、加强肠道屏障、刺激和调节免疫系统等^[5-8]^。这些功能大多与人类生理功能紧密相连。值得注意的是，除了细菌外，广义的肠道菌群还应包含其他关键微生物，如古细菌、病毒、噬菌体和真菌^[9]^。这些微生物可能通过控制肠道细菌的活动，间接发挥控制宿主的作用，其功能与细菌一样重要。

肠道菌群从人类的早期生命阶段开始就在宿主健康的不同方面具有潜在影响，肠道菌群和宿主之间的共生关系是通过一个复杂的相互作用网络来调节和稳定的，这个网络包括新陈代谢、免疫和神经内分泌之间的相互作用^[10]^。实现这种相互作用一方面与肠道上皮细胞有关，肠道上皮细胞除了具有屏障功能，还可以将肠腔内菌群产生的关键信息传递给位于固有层的免疫细胞。这主要是由具有模式识别受体（pattern recognition receptors, PRRs）的先天免疫系统完成的，如Toll样受体（Toll-like receptors, TLRs）和NOD样受体（Nod-like receptors, NLRs）^[11]^。这些受体共同识别病原体相关分子模式（pathogen-associated molecular patterns, PAMPs）（如脂多糖、肽聚糖和鞭毛蛋白）或受损组织中危险相关的分子模式。因此，肠道不仅包含了绝大多数驻留在人体中的微生物，而且还可能因此包含了人体中最大的免疫细胞库，从而对宿主整体具有免疫调节作用^[12]^；另一方面，这种串扰可能是由肠道菌群合成的具有多效性的代谢产物介导的^[13]^，包括叶酸、吲哚、次级胆汁酸、还有神经递质（如5-羟色胺、γ-氨基丁酸）和短链脂肪酸（short-chain fatty acids, SCFAs），这些代谢产物可以在生理上连接肠道和其他器官系统。

微生物群与宿主形成复杂的“超有机体”，其共生关系在生活的许多关键方面为宿主带来了好处，但也有越来越多的证据表明微生物群在致癌机制中也起着关键作用。研究^[14, 15]^表明，肠道菌群中如核梭杆菌、大肠杆菌、脆弱拟杆菌以及变形杆菌都与致癌有关。在肺癌患者中，肠道微生物群的组成往往也与正常人群有显著差异，Zhang等^[16]^通过对16S rRNA基因的V1-V2高变区测序首次揭示了肺癌患者肠道菌群的特征，发现在门水平上，肺癌人群与健康人群相比，拟杆菌门、梭菌门、蓝藻门、螺旋体门和黏胶球形菌门的丰度显著升高，而厚壁菌门和疣微菌门的丰度显著降低；在属水平上，两组间8个优势属也存在显著差异，肺癌组的拟杆菌属、韦荣球菌属、梭杆菌属丰度高于健康组，而志贺氏杆菌属、克吕沃尔氏菌属、普氏杆菌属、肠杆菌属、小杆菌属等丰度则均低于健康对照组。此外，部分肠道菌群代谢产物也与癌症的发生发展相关，研究^[17]^表明，SCFAs中的丙酸盐就具有调节免疫细胞产生抗微生物因子的作用，可作为免疫调节剂，包括在减少癌细胞增殖等方面发挥作用。而同属SCFAs的丁酸盐除了作为结肠上皮细胞的主要能量供给物质，在维持肠道内稳态方面挥着重要作用之外^[18]^，丁酸盐作为一种潜在的组蛋白去乙酰化酶抑制剂（histone deacetylase inhibitor, HDACI）还具有很强的抗癌活性^[19]^。研究^[20]^发现在非小细胞肺癌（non-small cell lung cancer, NSCLC）患者的肠道中存在明显的丁酸盐生产菌丰度下降的现象，这种变化或可通过“肺-肠轴”^[11]^对远端肺部的免疫反应起到相应的反馈作用。

肠道菌群丰度的改变与宿主健康的相关性虽然存在一定的个体差异性，但考虑到肠道菌群功能的多样性，这也使它仍旧是针对多种慢性疾病研究的焦点，如肺癌的防治研究。

## 2 联系机制

在中国，肺癌是最常见的癌症，发病率和死亡率多年居中国癌症类型中的首位，据国家癌症中心数据^[21]^显示，2015年我国新发肺癌病例约为78.7万例，发病率为57.26/10万，占当年癌症总发生率的20.03%；肺癌死亡人数约为63.1万例，死亡率为45.87/10万，占当年癌症总死亡率的26.99%。众所周知，香烟烟雾是肺癌的主要环境危险因素，原因之一是烟草中的致癌物造成了DNA损伤后突变与错误复制的增加^[22]^。此外，不良的饮食习惯、慢性炎症、电离辐射以及职业暴露（如石棉、铬、砷、二氧化硅和多环芳烃的长期接触）等都会增加肺癌发生的风险^[23]^。近年来的研究^[24-26]^表明肺癌的发生发展还与人体肠道菌群之间存在关联，这种相互作用可能与多种途径有关，如代谢、炎症或免疫途径。

### 2.1 影响代谢及化疗疗效

细胞代谢方式的变化可以促使肿瘤细胞的转化，而肿瘤细胞产生后也会重编其营养物获取和代谢的途径，以满足其自身所需的生物能、生物合成和氧化还原的需求，其表现形式可以是以下的一种或多种：对葡萄糖和氨基酸的摄取失调、使用机会性营养获取模式、使用糖酵解/三羧酸循环中间体进行生物合成和还原型辅酶Ⅱ（nicotinamide adenine dinucleotide phosphate, NADPH）生产、对氮的需求增加、代谢驱动的基因调控的改变以及与微环境的相互作用，这些重编的代谢途径现在被认为癌症的新兴标志之一^[27, 28]^。Liu等^[29]^通过KEGG（http://www.kegg.jp/或http://www.genome.jp/kegg/）和COG（http://www.ncbi.nlm.nih.gov/COG/）生物系统数据库分析表明，与健康群体相比，肺癌人群肠道微生物组中糖类的转运和代谢功能基团较少，并且也有着与Zhang等^[16]^同样的发现，即肺癌患者肠道内的厚壁菌群数量及厚壁菌/拟杆菌比率均下降明显，而厚壁菌群在人体能量代谢过程中起着重要的作用，它能将未消化的碳水化合物和蛋白质转化为乙酸，进而为生命活动提供能量。肠道厚壁菌群丰度的下降，会影响人体的果糖和甘露糖代谢、半乳糖代谢、戊糖和葡萄糖醛酸相互转化、淀粉和蔗糖代谢以及磷酸戊糖途径。这些发现提示了肠道菌群的变化改变了肺癌中的能量代谢途径。而Zheng等^[30]^在随后的研究中进行了更大范围的肺癌患者肠道菌群代谢途径的研究，他们发现与健康组相比，肺癌组中与细胞抗原、类固醇生物合成、泛素系统、转录相关蛋白、胆汁分泌和线粒体脂肪酸延伸相关的代谢通路增加，而与细菌运动蛋白、细菌趋化性、黄酮和黄酮醇生物合成、凋亡和G蛋白偶联受体相关的通路减少。这些数据表明在肺癌发展过程中肠道菌群确实存在新陈代谢的重新编程。

同时，肠道菌群代谢功能的差异，也会影响到肺癌的治疗效果。Song等^[31]^通过前瞻性研究发现，在接受程序性细胞死亡-1（programmed cell death 1, PD-1）抑制剂治疗的NSCLC患者中，与无进展生存期（progression‐free survival, PFS）≥6个月组相比，在PFS < 6个月组中，甲烷代谢、苯甲酸降解、二甲苯降解和真核生物核糖体合成等代谢途径明显下调；而鞭毛装配、细菌趋化、半乳糖代谢和钙信号传导等代谢途径明显上调。而具体到癌症的化学药物疗法中，其对临床治疗的影响可能主要表现在三个方面：促进药物疗效、削弱抗癌作用以及药物毒性的间介作用^[32]^。浙江大学的一项研究^[33]^表明，在Lewis肺癌小鼠模型中，使用顺铂联合ABX（万古霉素、氨苄青霉素和新霉素的抗生素混合物）治疗后，由于ABX破坏了肠道共生菌群的稳态，小鼠的肿瘤尺寸大于仅用顺铂治疗的小鼠，并且小鼠的存活率显著降低。而相较于顺铂单独使用，在顺铂联合乳酸杆菌治疗后，小鼠肿瘤显著减小，存活率更高。然而，肠道菌群对于化疗药物的作用并不总是具有积极影响，微生物对于药物的代谢作用可能导致严重的副作用，常常导致剂量限制，从而降低抗癌治疗的效果，甚至有时必须提前终止用药。例如，伊立替康诱导的黏膜炎症反应会导致30%左右的患者出现严重的剂量限制性腹泻^[34]^，这与伊利替康的水解产物SN-38在肠道再次被菌群产物β­葡萄糖醛酸酶激活产生肠毒性有关^[35]^。总之，肠道菌群通过介导药物的代谢反应，发挥了对化疗药物在癌症治疗中的调控作用。这些代谢反应除了影响较为广泛的还原和水解反应，还包括官能团去除、氮氧化物裂解、蛋白水解、脱硝、去共轭、胺形成和/或水解、噻唑开环、乙酰化和异恶唑断裂等^[36]^。但是在肺癌治疗过程中，肠道菌群与其代谢功能也受多种因素影响，例如宿主环境和饮食、手术干预、辅助药物（如抗生素）以及所用化疗药物。这些因素都可能会引起菌群及代谢失调。因此，虽然肠道菌群及其代谢功能的改变与肺癌存在相关性，但对于明确肺癌是这些改变的产物，还是这些改变是肺癌进展的正常结果，现在仍存在争议。

### 2.2 炎症与肺癌风险

炎症微环境在肿瘤的发生、发展，以及肿瘤治疗的敏感性中起重要作用，是癌症微环境的重要组成部分^[37]^。临床和流行病学研究^[38]^表明慢性感染、炎症和癌症之间有很强的相关性。例如，香烟烟雾和其他刺激性气体在引发肺部慢性炎症的过程中也起到了肺癌促进剂的作用，如慢性阻塞性肺病便是肺癌的高风险因素之一。同时，肺癌患者也经常发生局部感染（例如病毒感染、肺炎或肺结核）和炎症^[39]^。目前普遍认为，癌症相关的炎症细胞（包括先天免疫细胞和适应性免疫细胞）及其产生的多种细胞因子，如趋化因子、活性氧、基质金属蛋白酶（matrix metalloproteinases, MMPs）和干扰素等^[40]^共同形成了肿瘤炎症微环境，介导了肿瘤的发生和转移，并对肿瘤多种能力的形成起着重要作用。例如MMPs参与了癌症进展的所有阶段，包括肿瘤增殖、黏附、迁移、分化、血管生成、衰老、自噬、凋亡、免疫逃逸、侵袭或转移等^[41]^。目前，肿瘤炎症微环境已被认为是影响肺癌患者治疗和预后的重要因素。

人体长期暴露于各种微生物所构成的微环境中并受其影响，肠道微环境的稳态受肠道菌群及肠道屏障共同的调控。其中，肠道杯状细胞分泌的肠道黏液与潘氏细胞（paneth cell）产生的抗微生物肽有助于跨肠道黏膜界面分离宿主和微生物，构成了狭义的肠道屏障（广义肠道屏障还应包括肠道上皮层及其下由结缔组织构成的固有层）^[42]^。肠道菌群与肠道屏障之间稳定的动态平衡至关重要，可以有效增强宿主防御机制，避免受共生细菌、病原体和外来抗原的侵害，减轻、减少炎症反应^[43]^。肠道持续的炎症反应与肺癌的发生发展存在一定的相关性，炎症导致的肠道屏障的破坏会触发某些模式识别受体（如TLRs）^[44]^并引起随后的信号级联反应，如激活核因子κΒ（nuclear factor kappa-B, NF-κB）信号传导通路，NF-κΒ是癌症相关炎症的主要调节通路之一。在肺癌中，NF-κB主要通过介导炎性细胞因子分泌来建立炎性微环境促进肺癌的发生^[14, 45]^。

肠道微环境稳态的失衡也可以通过部分炎性因子循环增量的增加，使发生在肠道的炎症可以影响到远离肠道部位（如呼吸道）疾病的发展。Bindels等^[46]^发现在白血病小鼠中，肠道屏障的破坏及促炎细菌的移位引起了全身炎症反应的增强，主要表现为血清脂多糖结合蛋白（serum lipopolysaccharide binding protein, sLBP）和白细胞介素6（interleukin 6, IL-6）水平的升高，这也是几种恶病质症状（肌肉萎缩、厌食和体重减轻）的主要驱动因素。这一发现在Bindels等^[46]^随后的前瞻性研究中得到了进一步验证，在大肠癌或肺癌患者中，sLBP水平低（sLBP < 18.25 μg/mL）的患者生存率比sLBP水平高（sLBP > 18.25 μg/mL）的患者高3倍，在以IL-6水平（5.7 pg/mL）为分层依据时，也有相似结论。同时，通过建立回归模型进行预测分析发现sLBP水平每增加一个单位，患者发生死亡、厌食和恶病质的几率就分别增加7%、9%和7%。Yang等^[47]^则发现，在从不吸烟的人群中，膳食纤维（益生元的主要来源）或酸奶（益生菌食品）的摄入量与肺癌发生的风险成负相关：最高摄入量人群肺癌的患病风险相较于最低摄入量人群降低了30%以上。这与益生元和益生菌在肠道中增加抗炎细胞因子IL-10、降低促炎细胞因子IL-1β和IL-6的分泌有关^[48]^，这也从另一个角度验证了Bindels等的结论。Zhang等^[49]^发现在大肠腺瘤和结直肠癌（colorectal cancer, CRC）患者中，血清中的炎性因子C反应蛋白（C-reactive protein, CRP）和肿瘤坏死因子受体2（serum tumour necrosis factor receptor‐2, sTNFRII）随着癌症恶性程度的增加呈顺序变化，并且CRP和sTNFRII的水平与CRC患者肠道中丰度增加的菌群（如*Peptostreptococcus stomatis*、*Parvimonas micra*、*Gemella morbillorum*等14种菌属）呈正相关，而与CRC患者肠道中丰度减少的菌群（如*Eubacterium eligens*、*Coprococcus comes*、*Eubacterium hadrum*等10种菌属）呈负相关。而Shiels等^[50]^则发现CRP（四分位数4 *vs* 1：OR=1.77，95%CI：1.23-2.54，*P*_trend_ < 0.001）和sTNFRII（OR=1.70, 95%CI: 1.18-2.45, *P*_trend_ < 0.006）的循环水平还与肺癌有显著的统计学相关性，但研究中也提及在无吸烟史的肺癌患者中，CRP和sTNFRII均与罹患肺癌的风险无关（*P*_trend_ > 0.05）。

### 2.3 调节免疫及其作用机制

免疫系统在肿瘤的发展过程中起着双重作用，它一方面可以在一个称为免疫监视的过程中识别和控制新生肿瘤细胞，另一方面也可以通过多种机制进行免疫抑制从而达到促进肿瘤细胞发展的作用，这一双重机制也被称为癌症免疫编辑（cancer immunoediting）^[51]^，一般认为癌症免疫编辑是一个动态过程，包括三个阶段：消除、平衡和逃逸^[52]^。在消除阶段，先天免疫系统和适应性免疫系统共同检测并消灭早期肿瘤；在平衡阶段，免疫系统使肿瘤处于功能性休眠状态，但部分肿瘤细胞可能会由于面临持续的免疫监视压力，发生遗传和表观遗传学的变化，从而进化出抵抗免疫识别的能力诱导免疫抑制；在逃逸阶段，肿瘤细胞具有了规避免疫系统识别的能力，肿瘤细胞获得了无限制增殖的条件，并形成了免疫抑制性肿瘤微环境^[53]^。根据肿瘤发展的这一特点，免疫疗法为肿瘤治疗提供了另一种可能，其策略包括给予促炎细胞因子以刺激免疫，使用单克隆抗体去除免疫检查点，以及使用癌症疫苗增强对肿瘤的免疫力^[54]^。目前，在部分晚期NSCLC患者的临床治疗中，免疫检查点抑制剂（immune checkpoint inhibitors, ICIs）之一的PD-1抑制剂确实表现出了持久的抗肿瘤活性^[55]^。

肠道菌群对于免疫系统的影响范围远超出了肠道局部，其对免疫系统的影响也涉及肺癌免疫治疗的疗效。研究表明肠道菌群多样性高的晚期NSCLC患者在接受尼伏单抗（nivolumab）治疗后较多样性低的患者反应明显较好，就PFS而言，肠道菌群多样性高的患者的中位PFS可达到209 d，而多样性低的患者仅有52 d，而这可能与肠道菌群多样性高患者外周记忆T细胞和自然杀伤细胞的增多相关^[56]^。但不同的肠道菌群对癌症的作用不尽相同，一些“坏菌群”能做为癌症的诱发因素，如前文所提及的核梭杆菌、大肠杆菌、脆弱拟杆菌以及变形杆菌等，而“好菌群”则能显著提高癌症的治疗的效果。实验发现，口服双歧杆菌可通过诱导树突状细胞功能并增加肿瘤微环境中CD8^+^ T细胞的积累来改善癌症小鼠模型中对抗程序性细胞死亡配体1（programmed cell death protein 1 ligand 1, PD-L1）抗体的反应^[57]^。“好菌群”的积极作用在临床研究^[58]^中也得到证实，在接受免疫检查点封锁（immune checkpoint blockade, ICB）治疗之前和/或之后进行丁酸梭菌疗法（Clostridium butyricum therapy, CBT），可显著延长晚期NSCLC患者的PFS和总生存时间（overall survival, OS）。接受过CBT治疗的晚期NSCLC患者中位PFS可达到250 d，而未接受过CBT治疗的晚期NSCLC患者中位PFS仅有101 d，中位OS则为361 d（接受CBT治疗的晚期NSCLC患者的中位OS未达到）。并且，即使在接受过抗生素治疗的晚期NSCLC患者中，CBT治疗也与更长的PFS和OS有显著关联。同时，Tomita等^[58]^与Gui等^[20]^的发现也在一定程度上说明部分肠道菌属与肺癌的发生发展确实存在联系。

目前，肠道菌群发挥肺部免疫调节作用的机制尚未完全明确，但可能涉及以下2种途径。第一是“肺-肠轴”^[11, 59, 60]^理论，肠道菌群及其产物被抗原呈递细胞吞噬并转移到肠系膜淋巴结，在那里刺激T和B细胞的活化。一旦激活，这些细胞就会表达某些趋化因子受体（如CCR4和CCR9）获得归巢特性，可以通过淋巴和血液循环迁移回到原始位置（肠黏膜）或远端位置，如气道^[61, 62]^。在那里，它们可以直接作用于目标或继续刺激其他免疫细胞；另一方面，来自肠道的菌群产物或活的菌群也可以直接通过血液或淋巴循环到达肺部，以刺激免疫系统。根据组织受到的刺激类型以及所处的免疫状态，结果可以是多样的，可能产生有效的抗炎或抗肿瘤活性，也可能进一步促进组织损伤、病原体定植和肿瘤进展。第二是“肠-骨髓”调节机制^[63, 64]^，肠道菌群的代谢产物是微生物相关分子模式（microbe-associated molecular patterns, MAMPs）和PAMP的来源，MAMP或PAMP通过结合诸如单核细胞、巨噬细胞和自然杀伤细胞等免疫细胞上的PRRs使其活化。同时，这些微生物来源的抗原通过血液循环到达骨髓，影响髓系免疫细胞的分化和功能，如诱导具有长期“记忆”特性的细胞的产生。Zitvogel等^[65]^认为这些来自肠道菌群的抗原和肿瘤抗原之间具有相似性，从而通过抗原模拟或交叉反应来激发免疫细胞的活性，并形成了相应的T细胞库，从而提高了免疫系统在识别癌细胞时的反应性和抗肿瘤能力，即增强免疫监视能力。此外，Dang等^[66]^发现肠道菌群产物中的SCFAs可以通过促进骨髓造血前体的生成，发挥免疫调节的功能。

## 3 结语

肠道菌群对肠道局部和宿主整体健康的重要性正在被重新认识。近年来，肠道菌群的存在及其在肺癌中的作用也备受关注。肠道菌群即可以因为肺癌的发生发展、化疗药物等诸多治疗措施的干预而不断地被重新调控，也可以通过代谢、炎症和免疫反应等途径对肺癌发挥远端作用。目前的研究已经开始识别特定的菌群和分子途径，它们要么可以在肿瘤的发生过程中起抑制或促进作用，要么对现有的肿瘤治疗方案发挥促进或抑制的作用。虽然目前的研究表明单个微生物群体可能不足以致癌，而是需要宿主（如免疫系统）、环境（如膳食诱变剂）或其他微生物（增强效应）的共同作用才能发挥致癌效应^[67]^。但是由于肠道菌群的恢复力、稳定性及其对生理、病理和环境变化的反应性，使得靶向肠道菌群的肺癌的诊断、干预、治疗措施已具备一定可行性。因此，针对肺癌中的肠道菌群研究可能成为精准和个性化医学的下一个前沿领域之一。
